# A Case Report: A rare primary adrenal malignant rhabdoid tumor in a 42-year-old man

**DOI:** 10.3389/fonc.2026.1971354

**Published:** 2026-09-30

**Authors:** Jiali Zhu, Jingcui Guo, Yang Zhang, Lin Liu, Shengya Xu, Nan Gu, Ying Gao

**Affiliations:** Department of Endocrinology, Peking University First Hospital, Beijing, China

**Keywords:** case report, malignant rhabdoid tumor, primary adrenal neoplasm, SMARCB1 deletion, targeted therapy

## Abstract

**Background:**

Primary adrenal malignant rhabdoid tumor (MRT) is a rare, highly aggressive neoplasm lacking specific clinical manifestations and imaging characteristics.

**Methods:**

This case report delineates the clinical course of a patient with primary adrenal MRT managed at our institution. We provide a comprehensive characterization of the patient’s clinical presentation, histopathological and molecular genetic profiles, and the therapeutic strategy employed. Furthermore, we critically examine contemporary evidence regarding its pathogenesis, diagnostic challenges, and therapeutic strategies.

**Results:**

A 42-year-old man was initially admitted to the Department of Endocrinology presenting with pyrexia and an adrenal mass. The patient underwent surgical resection of an adrenal mass; definitive histopathological examination and molecular genetic profiling confirmed a diagnosis of primary adrenal MRT. Despite undergoing surgical resection and adjuvant chemotherapy, the patient ultimately succumbed to sudden cardiac death.

**Conclusion:**

Primary adrenal MRT represent an exceptionally rare clinical entity. Consequently, clinicians must maintain high suspicion for this diagnosis when evaluating non-functioning adrenal masses, especially those with atypical radiological features or aggressive behavior.

## 1 Introduction

Malignant rhabdoid tumor (MRT), first reported in 1978, is a rare, highly aggressive neoplasm primarily affecting infants and children under three years (1). Primary adrenal MRT is exceptionally rare, with only 4 reported cases (2–5). It lacks specific clinical manifestations and patients may present with abdominal pain, fatigue, or malaise, complicating early diagnosis and increasing misdiagnosis. Definitive imaging features are absent, thus histopathological examination and molecular genetic testing are particularly important for diagnosis. Biallelic deletion of SWI/SNF-related matrix-associated actin-dependent regulator of chromatin subfamily B member 1 (SMARCB1) is the hallmark genetic alteration associated with MRT (6). The SMARCB1 protein, a core subunit of the SWI/SNF chromatin remodeling complex, regulates pathways critical for cellular differentiation and development (7). Histology typically reveals round/polygonal cells with eccentric nuclei, prominent nucleoli, and eosinophilic cytoplasm. Immunohistochemical analysis demonstrates strong vimentin positivity and frequent EMA/cytokeratin expression; integrase interactor 1 (INI1) shows complete nuclear loss (6). We present a case of primary adrenal MRT in an adult, delineating its clinical, pathological, and genetic profiles alongside diagnostic and therapeutic management strategies.

## 2 Case presentation

In October 2023, a 42-year-old man presented with a left adrenal mass and fever. A 2.58 × 2.14 cm lesion, initially identified incidentally on routine chest computed tomography (CT) scan in March 2023, was not evaluated. Subsequently, the patient experienced intermittent limb weakness, back pain, and ankle joint gout. Beginning in September 2023, he exhibited daily fevers (37.5–38.5 °C) with progressive constitutional symptoms, unresponsive to empirical antibiotics. Throughout the disease course, the patient maintained normotensive status.

His laboratory tests revealed a white blood cell count of 10.2 (normal range: 3.5-9.5) ×10^9^/L, hemoglobin of 119 (normal range: 130-175) g/L, platelet count of 514 (normal range: 120-350) ×10^9^/L, neutrophil percentage of 76.4 (normal range: 40.0-75.0) %, lymphocyte percentage of 15.4 (normal range: 20.0-50.0) %, erythrocyte sedimentation rate of 106 (normal range: 0-15) mm/h, interleukin-6 concentration of 34.7 (normal range: < 6.4) pg/ml, and high-sensitivity C-reactive protein level of 133.65 (normal range: 0.00-3.00) mg/L. Serum neuron-specific enolase (NSE) was elevated at 16.80 (reference range: <16.3) ng/ml. Procalcitonin and other tumor markers were unremarkable.

To characterize the functional status of the left adrenal mass, a comprehensive endocrine workup was performed. This included: serum cortisol and adrenocorticotropic hormone (ACTH) circadian rhythm, 24-hour urinary free cortisol, 1 mg overnight dexamethasone suppression test, upright plasma renin activity and aldosterone and captopril challenge test, adrenal sex steroid panel (dehydroepiandrosterone sulfate [DHEA-S], androstenedione, testosterone), plasma catecholamines and their metabolites (norepinephrine, epinephrine, dopamine, metanephrine[MN], normetanephrine[NMN], 3-methoxytyramine[3-MT]). All results were within normal ranges, confirming that the adrenal mass was non-functional.

Contrast-enhanced CT and magnetic resonance imaging (MRI) in October 2023 revealed a left adrenal mass measuring approximately 6.4 × 5.4 × 6.2 cm. The MRI showed heterogeneous enhancement of the mass with left renal vein filling defects (**Figure 1**). Positron Emission Tomography (PET)-CT findings included an irregular nodule in the lateral segment of the right middle lobe with increased glucose metabolism, a soft tissue mass in the left adrenal gland with elevated glucose metabolism, and a probable venous tumor thrombus, all highly suggestive of a malignant process. Enlarged lymph nodes around the pancreas and abdominal aorta, some with increased glucose metabolism, were considered potential metastases. Additionally, there were multiple lymph nodes in the neck, axilla, inguinal region, and near iliac vessels, along with enhanced bone and spleen metabolism, which were more consistent with inflammation. Iodine-131 metaiodobenzylguanidine (MIBG) scintigraphy demonstrated no abnormal uptake. These collective findings indicated malignant adrenal neoplasia.

**Figure 1:**
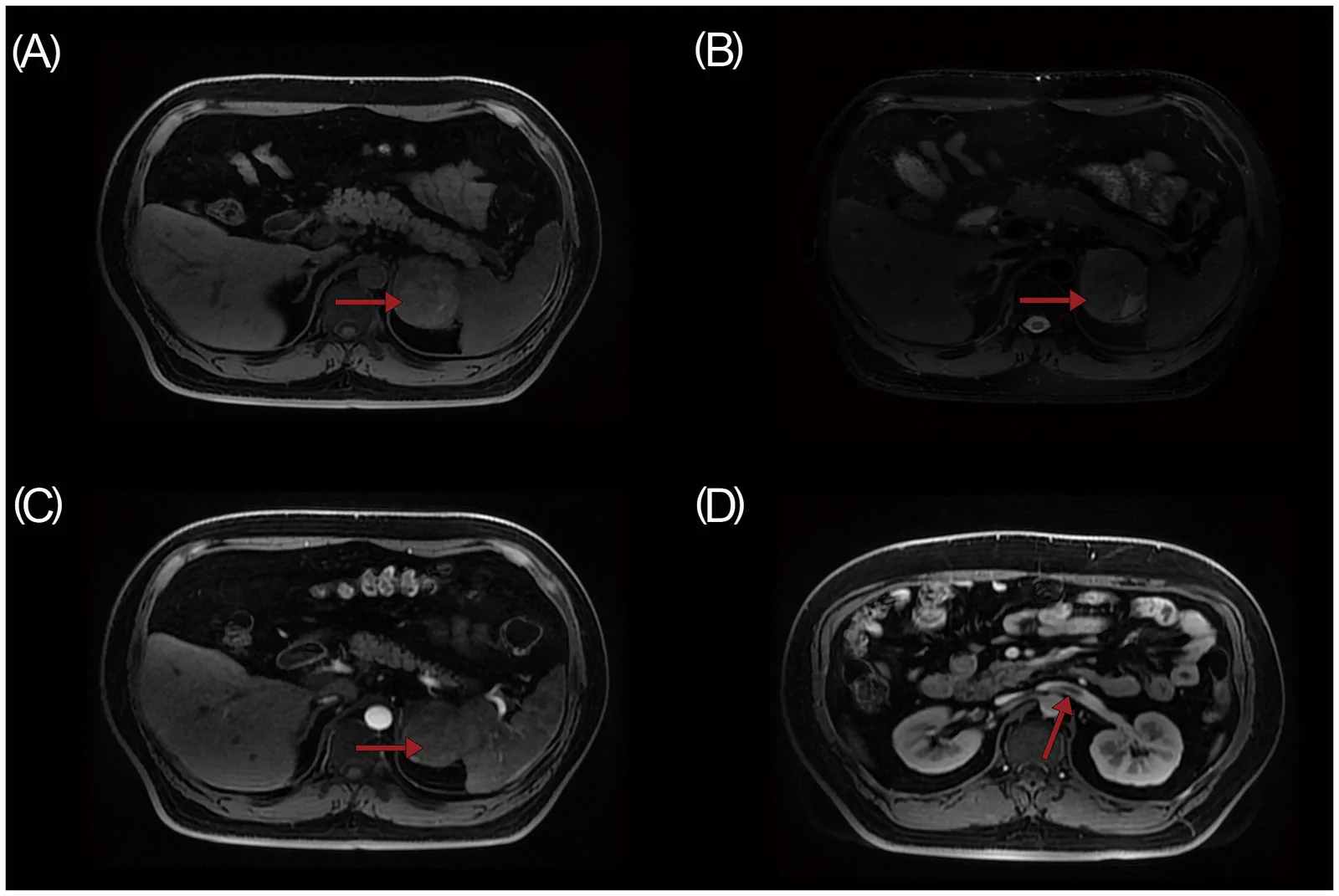
**(A)** Contrast-enhanced adrenal MRI showed a 6.4×5.4×6.2 cm left adrenal mass with heterogeneous T1-weighted signal. **(B)** Heterogeneous hyperintensity on T2-weighted imaging. **(C)** Heterogeneous post-contrast enhancement. **(D)** Filling defects in the left renal vein.

Regarding additional preoperative assessments, transthoracic echocardiography demonstrated normal left ventricular ejection fraction (LVEF > 55%), no valvular abnormalities, and no pericardial effusion. A 12-lead electrocardiogram (ECG) showed normal sinus rhythm without ST-T segment changes, conduction abnormalities, or QTc prolongation. The patient had no history of cardiovascular disease, hypertension, diabetes, or dyslipidemia.

Preoperative multidisciplinary discussion concluded that the left adrenal mass represented the dominant disease requiring urgent surgical management. A two-stage strategy was formulated: Stage 1 – left adrenalectomy with thrombectomy and lymphadenectomy for local control and histopathological diagnosis; Stage 2 – subsequent management of the right pulmonary nodule (wedge resection, stereotactic radiotherapy, or systemic therapy) guided by the pathological findings. Resection of left adrenal mass was performed in November 2023. During surgical exploration, the adrenal was entirely replaced by tumor. The lesion adhered to the pancreatic tail and peri-renal fascia, with a solid thrombus extending into the left renal vein. No gross invasion of bowel or ureter was noted; the kidney and pancreas were preserved. The procedure comprised en bloc resection of the left adrenal tumor, thrombectomy of the left renal vein, and dissection of para-aortic and peripancreatic lymph nodes. Histopathological examination confirmed negative surgical margins. Histopathology (H&E; **Figures 2A, B**) and immunohistochemistry confirmed the diagnosis of MRT. The tumor exhibited positivity for epithelial membrane antigen (EMA), Cytokeratin AE1/AE3, CD99, NSE and synuclein; variable expression of vimentin and inhibin; negativity for INI-1, Melan A, SF-1, MyoD1, chromogranin A, smooth muscle actin, CD34, Paired Box 8, S-100 protein and ERG. Whole-exome sequencing of the tumor tissue confirmed a SMARCB1 deletion (**Figures 2C**), which was consistent with the molecular profile characteristic of MRT.

**Figure 2:**
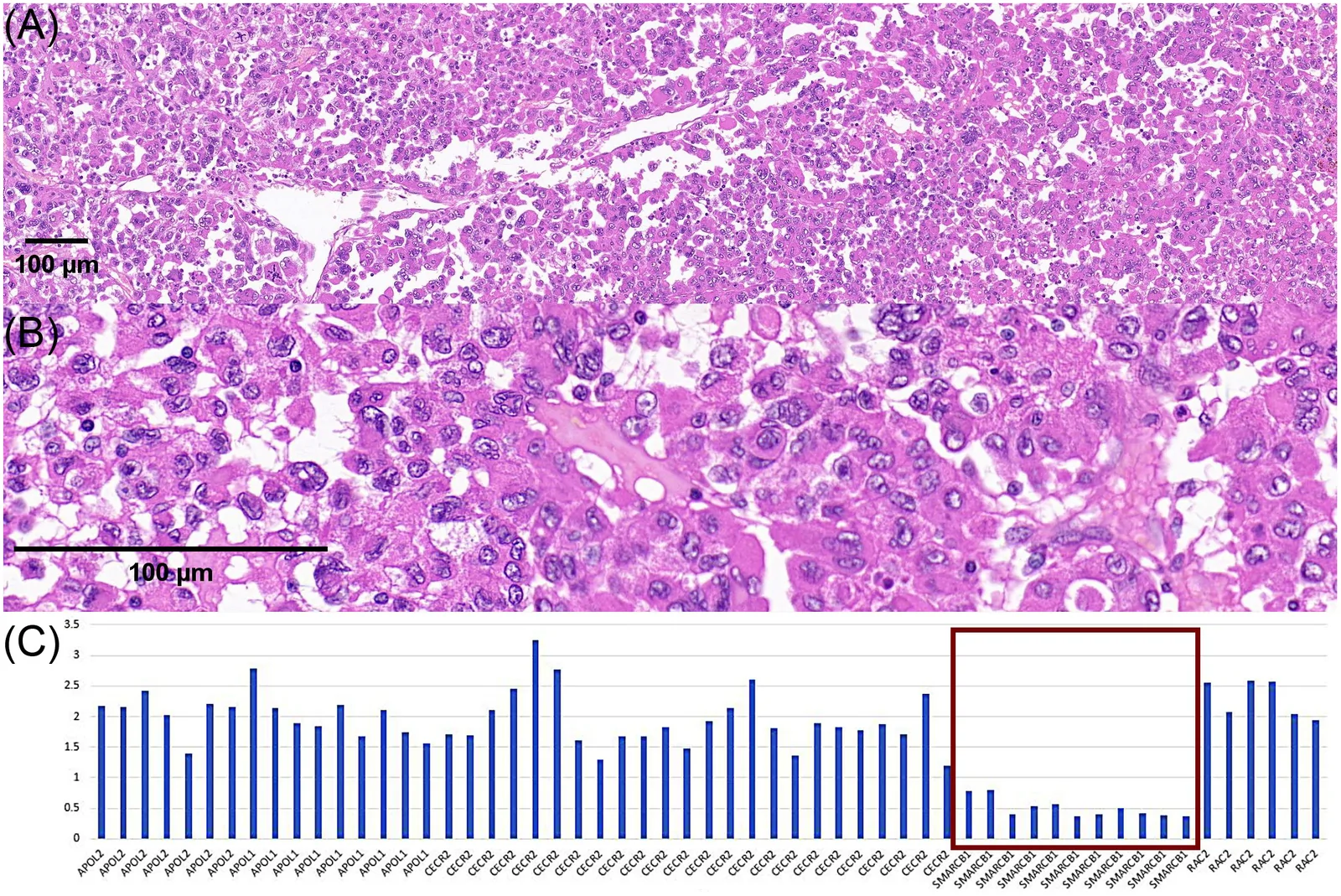
[**(A)** ×100; **(B)** ×400] H&E staining showed rhabdoid cells with abundant eosinophilic cytoplasm, perinuclear inclusions, and eccentric vesicular nuclei. **(C)** SMARCB1 deletion confirmed by whole-exome sequencing.

Following surgery, the patient’s body temperature normalized. The January 2024 CT scan showed postoperative changes in the left adrenal region, with low-density and metallic areas. It also revealed low-density changes around the left kidney, mild peritoneal fluid, patchy densities in the left abdominal wall, multiple retroperitoneal and iliac lymph nodes, and a slightly enhanced 1.1 × 1 cm nodule in the right middle lung lobe.

The patient received treatment with Anlotinib from January to March 2024. The March 2024 CT scan revealed multiple heterogeneously enhanced nodules and mass-like lesions in the postoperative left adrenal region, perirenal space and fascia, splenic hilum, paraesophageal area, and retroperitoneum, with the largest measuring 4.5 × 4 cm. The lesions showed indistinct margins with adjacent structures, including the diaphragm, spleen, left kidney, left renal vein, and bowel. Multiple osteolytic lesions with soft tissue masses were also observed, suggesting bone metastases. Collectively, these findings were consistent with progressive disease (PD).

Treatment with tazemetostat was administered from March to June 2024. The June 2024 CT scan showed a persistent solid nodule in the right middle lung lobe, consistent with metastasis. Bone metastases demonstrated a mixed response, with both regression and progression, notably in the left fifth rib and manubrium. Multiple metastatic lymph nodes in the abdomen, pelvis, and retroperitoneum showed variable size changes. A new cystic lesion was identified posterior to the left psoas muscle, and the metastasis at the left pubic symphysis had enlarged. The treatment response was assessed as PD.

From June to September 2024, the patient was treated with a combination regimen of Dacarbazine and Doxorubicin Liposome (AD) for four cycles. The September 2024 CT scan indicated a mixed response (overall stable disease [SD]/PD), with some lesions increasing in size, others remaining stable, and a few showing reduction. Following, the patient was advised to try HH-2853 (dual target inhibitor of EZH2), in conjunction with palliative radiotherapy for symptomatic bone metastases. However, the patient declined further aggressive therapeutic interventions and opted for best supportive care alone. In December 2024, the patient experienced a sudden cardiac arrest at home; despite immediate resuscitation efforts, these proved unsuccessful, resulting in death. As no post-mortem examination was conducted, the precise etiology of death remains undetermined. The timeline of treatment for this case is shown in **Figure 3**.

**Figure 3:**
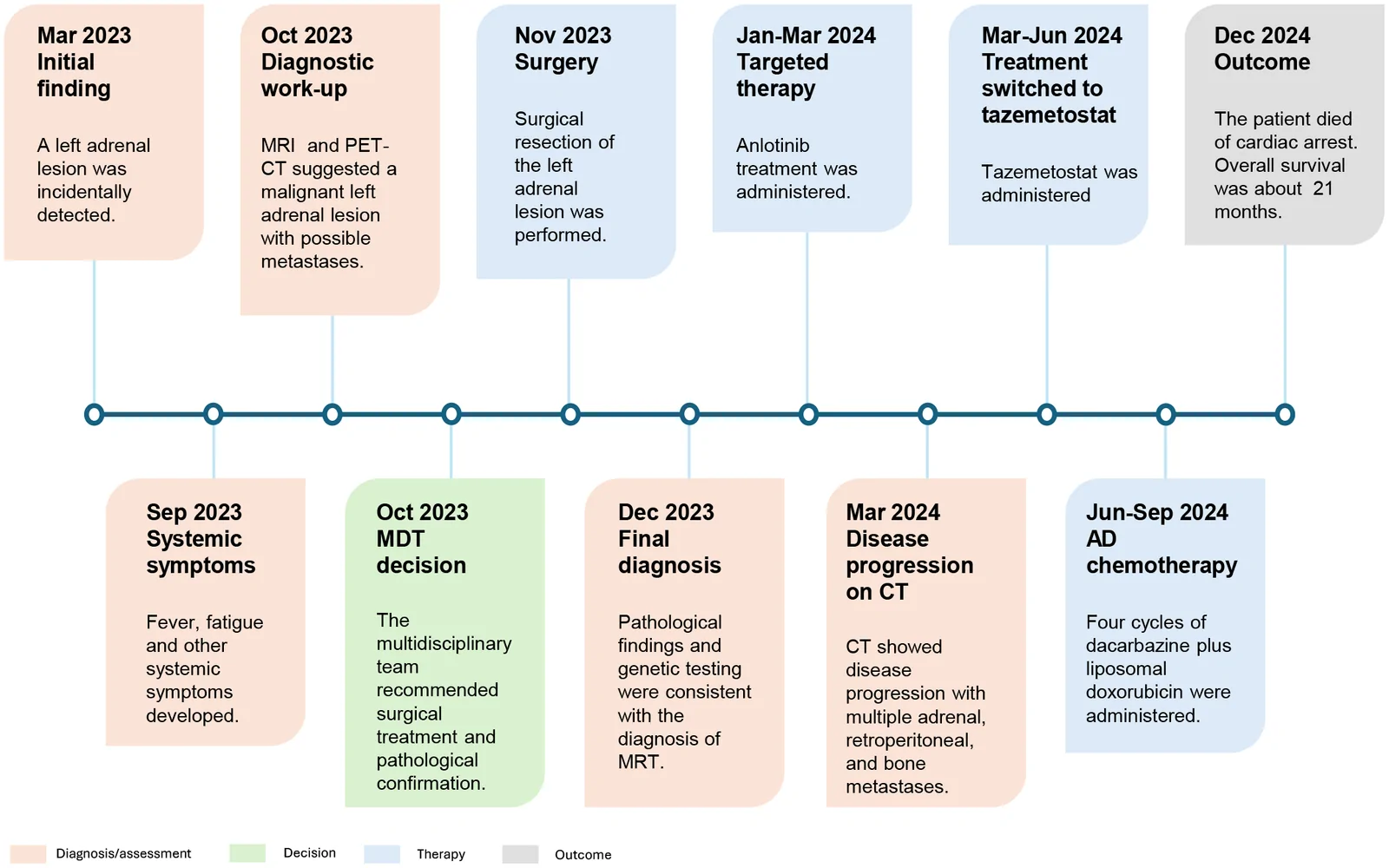
Clinical course and treatment timeline of the patient.

## 3 Discussion

Malignant rhabdoid tumor (MRT) is a rare, highly aggressive neoplasm that predominantly affects infants and young children. Anatomically, MRTs arise most frequently within the central nervous system (approximately 65%), followed by the kidneys (9%) and other extracranial sites (26%) (1). Primary adrenal involvement is exceptionally rare. To the best of our knowledge, this is the second report of primary adrenal MRT in an adult male. The patient presented with persistent low-grade fever, and the imaging and pathological findings were largely consistent with those described in previous reports. Despite comprehensive multimodal management comprising surgical resection, systemic chemotherapy, and targeted therapy, the patient exhibited disease progression. This refractory clinical course underscores the aggressive clinical behavior and unfavorable prognosis of this malignancy. Previously reported cases of primary adrenal MRT and the case described in the present study are summarized in **Table 1**.

**Table 1:** Summary of previously reported cases of primary adrenal malignant rhabdoid tumor and the present case.

Study	Age/Sex	Tumor size	Imaging findings	histopathological features	immunohistochemical/molecular findings	Treatment	Outcome
Yaris et al., 2002 (5)	3/M	10 × 10 cm	Large adrenal mass invading the left diaphragm	Pleomorphic tumor cells with prominent nucleoli and eosinophilic cytoplasmic inclusions	INI: NA, Vimentin: + (strongly), EMA: +, CK: + (weak), S-100: −, CD34: NA	Subtotal resection + chemotherapy	Rapid progression and death
Shim et al., 2014 (4)	63/M	13 × 12 × 8 cm	Large adrenal mass without adjacent organ invasion	Polygonal cells with eosinophilic cytoplasmic inclusions	INI: NA, Vimentin: −, EMA: −, CK: +, S-100: −, CD34: −	Laparoscopic resection	No recurrence or metastasis for >2 years
Al-Daghmin et al., 2019 (3)	21/F	10 × 8 × 6 cm	Adrenal mass with retroperitoneal and retrocrural lymph node and pulmonary metastases	Polygonal rhabdoid cells with abundant eosinophilic cytoplasm, prominent nucleoli, extensive necrosis, and high mitotic activity	Complete INI loss, Vimentin: +, EMA: NA, CK: +, S-100: NA, CD34: NA	Neoadjuvant radiotherapy + chemotherapy + adrenalectomy	No recurrence or metastasis for >1 year
Alturkustani et al., 2022 (2)	14/F	8.0 × 8.0 × 6.5 cm	Heterogeneous adrenal mass without invasion of adjacent organs	Predominantly small round blue cells with focal rhabdoid cells; rosette, glandular, and chondroid differentiation; necrosis and hemorrhage	Complete SMARCB1/INI1 loss, Vimentin: NA, EMA: +, CK: +, S-100: +, CD34: − biallelic SMARCB1 frameshift mutations	Surgical resection	Not reported
Present case	42/M	6.4 × 5.4 × 6.2 cm	Adrenal mass with peripancreatic and para-aortic lymph node and pulmonary metastases	Polygonal rhabdoid cells with abundant eosinophilic cytoplasm, eccentric vesicular nuclei, and prominent nucleoli	Complete INI1 loss, Vimentin: +/−, EMA: +, S-100: −, CD34: − SMARCB1 deletion	Surgical resection + chemotherapy + targeted therapy	Survival for approximately 1 year and eventually death

M, male; F, female; EMA, epithelial membrane antigen; CK, cytokeratin, INI1, integrase interactor 1; CD34, cluster of differentiation 34; S-100, S-100 protein; NA, not available; −, negative; +, positive; SMARCB1, SWI/SNF-related matrix-associated actin-dependent regulator of chromatin subfamily B member 1.

No established therapeutic protocol exists for primary adrenal MRT. All four reported cases underwent surgery; two survived for more than 2 years postoperatively, while one succumbed during initial multidrug chemotherapy. The EU-RHAB recommends multimodal therapy combining surgery, chemotherapy with radiotherapy for all MRT patients. This approach achieves 3-year overall and event-free survival rates of 39.3% and 32.3%, respectively (8). Prognostic determinants include age, metastatic stage, germline mutations, and DNA methylation subgroups (9). Nevertheless, extracranial MRTs continue to be associated with a dismal prognosis, particularly in the pediatric population. A recent systematic review and meta-analysis encompassing 398 pediatric cases revealed a pooled metastatic rate at diagnosis of 41.4% and an overall mortality rate of 68.7%, notwithstanding the widespread utilization of aggressive multimodal therapies, including intensive chemotherapy and surgical resection (10). These findings highlight the highly aggressive biological behavior of MRT and indicate that current multimodal treatment strategies remain insufficient to substantially improve survival. Novel inhibitors targeting zeste homolog 2 (EZH2), DNA methyltransferases and histone deacetylases constitute promising investigational strategies (11).

In this case, the initial treatment phase involved the administration of anlotinib, a multi-targeted tyrosine kinase inhibitor (TKI) approved in China for soft tissue sarcoma following the failure of standard therapeutic regimens. Given the vascular-rich nature of MRT and the lack of established targeted therapy for SMARCB1deficient tumors, anlotinib was selected as an empirical first-line anti-angiogenic agent. Subsequent administration of tazemetostat, an EZH2 inhibitor specifically approved by the U.S. Food and Drug Administration (FDA), which is indicated for the treatment of epithelioid sarcoma characterized by SMARCB1/INI-1 deficiency. Given that MRT shares the same SMARCB1-deficient molecular mechanism, tazemetostat represents a biologically rational targeted therapy. However, its efficacy in extra-cranial MRT (especially adult adrenal primary) has not been established, and our case confirms limited activity. Finally, the combination of dacarbazine and liposomal doxorubicin represents the standard-of-care regimen for the management of advanced soft tissue sarcoma. Liposomal doxorubicin was preferred over conventional doxorubicin to reduce cardiotoxicity, especially given the patient’s prior exposure to anti-angiogenic therapy and the risk of cumulative cardiac stress. The mixed response observed is consistent with the generally chemoresistant nature of MRT. Despite multimodal antitumor therapy, our patient succumbed 12 months postoperatively.

Our case report is subject to several notable limitations. First, following a multidisciplinary team consultation, the decision was made to proceed directly to definitive surgical resection of the primary adrenal mass for both diagnostic clarification and therapeutic intervention. While preoperative biopsy of the pulmonary nodules could have facilitated earlier confirmation of synchronous metastasis, it was omitted to avoid delaying definitive surgery. Second, regarding the right lung lesions, stereotactic body radiotherapy (SBRT) was initially planned in the postoperative setting. However, upon the confirmed diagnosis of MRT, systemic therapy was prioritized, resulting in the deferral of SBRT. Although palliative radiotherapy was recommended for symptomatic bone metastases, the patient declined further treatment interventions. Finally, despite the absence of any documented history of cardiovascular disease, the patient experienced sudden cardiac arrest leading to death. As a postmortem examination was not performed, the precise etiology of death remains undetermined.

In conclusion, primary adrenal MRT exhibits nonspecific clinical and imaging features. Upon detection of an adrenal mass, timely evaluation and follow-up are essential. Pathological and genetic testing, along with early intervention, are paramount for diagnosis and prognosis.

## 4 Patient perspective

A direct patient perspective could not be obtained because the patient had died.

## Data Availability

The original contributions presented in the study are included in the article/supplementary material. Further inquiries can be directed to the corresponding author.
